# Surgical and medical management of an oral spindle cell sarcoma in an African hedgehog (*Atelerix albiventris*)

**DOI:** 10.1002/vms3.738

**Published:** 2022-01-25

**Authors:** Andrew Lacqua, Rebecca Dreizen, Peter Helmer

**Affiliations:** ^1^ Surgery Service BluePearl Pet Hospital Tampa Florida USA; ^2^ Friendship Animal Hospital Washington District of Columbia USA; ^3^ Avian and Exotics Service BluePearl Pet Hospital Clearwater Florida USA

**Keywords:** hemimandibulectomy, neoplasia, small mammal, soft tissue sarcoma, spindle cell tumour

## Abstract

A 2.5‐year‐old male African hedgehog (*Atelerix albiventris*) presented with an intraoral, soft tissue mass overlying the left mandible. A computed tomography scan and subsequent fine needle aspirate suggested a malignant spindle cell tumour. The tumour was excised with a partial hemimandibulectomy. Histopathology demonstrated an incompletely excised spindle cell sarcoma. The hedgehog underwent adjuvant therapy consisting of intravenous carboplatin and oral lomustine, followed by palliative radiation therapy once tumour recurrence was noted on follow‐up surveillance. Radiation therapy was initially successful in decreasing tumour size, but the hedgehog re‐presented a month later acutely non‐ambulatory paraparetic with a distal right antebrachial mass. Diagnostics including radiographs and fine needle aspirate were consistent with metastatic neoplasia and humane euthanasia was elected.

## Graphical abstract

A 2.5‐year‐old male African hedgehog (*Atelerix albiventris*) presented with an oral mass which, following a CT scan and cytology confirmed a soft tissue sarcoma. The oral soft tissue sarcoma was excised with hemimandibulectomy and treated with follow‐up chemoradiotherapy.

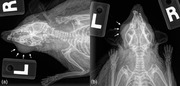

## 1 CASE DESCRIPTION

A 2.5‐year‐old intact, 290‐g male African Hedgehog (*Atelerix albiventris*) was presented for a bleeding oral mass. The mass was suddenly noticed by the owners who reported no prior medical history. Additional information regarding the origin of the hedgehog, housing, diet and husbandry is relevant to provide full history, although it was not ascertained in this case. The hedgehog was manually restrained and anaesthetized with sevoflurane gas administered via face mask. Physical exam revealed a 1.5 cm × 1.0 cm intraoral soft tissue mass overlying the left mandible.

**FIGURE 1:**
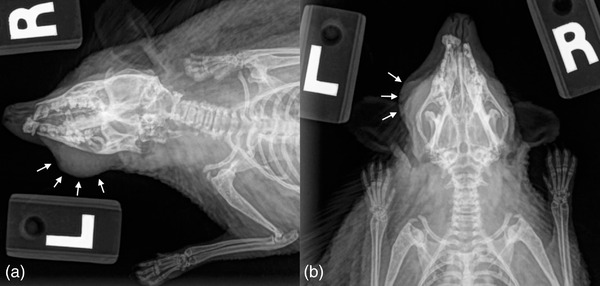
Initial right lateral (a) and dorsoventral (b) radiographs of the hedgehog. There is soft tissue swelling on the lateral aspect of the left maxilla and a displaced rostral maxillary fracture. Additionally, there is a radiolucent region within the caudal left hemimandible adjacent to a soft tissue mass with underlying focal cortical disruption

While anaesthetized, blood was collected from the cranial vena cava using a 25‐gauge needle and 1 ml syringe, full body radiographs and abdominal ultrasound were performed, and a fine needle aspirate of the mass was obtained. Bloodwork showed mild anaemia and elevated blood urea nitrogen and creatine phosphokinase likely secondary to the bleeding oral mass and the ingestion of blood (Table 1). Radiographs revealed a soft tissue mass overlying the left mandible with underlying osseous destruction and a displaced right rostral maxillary fracture (Figure 1). Abdominal ultrasound revealed no evidence of metastatic neoplasia or other abnormalities. Cytology of the aspirate showed bacterial rods, squamous epithelial cells and spindle cells, but no definitive signs of malignant neoplasia. However, given the presence of spindle cells and the likelihood of poor tumour cell exfoliation, a malignant spindle cell sarcoma was suspected. Pre‐operative computed tomography (CT) was recommended to plan for tumour excision with a partial left hemimandibulectomy.

**TABLE 1:** Initial and follow‐up hematologic and serum biochemical values of the hedgehog with adult reference intervals

Test	Initial results	Follow‐up results	Adult reference range (Helmer & Carpenter, 2018)
**Serum chemistry**			
Total Protein (g/dl)	5.6	6.2	4–7.7
Albumin (g/dl)	2.6	3.4	1.8–4.2
Globulin (g/dl)	3.0	2.8	1.6–3.9
A/G Ratio	0.9	1.2	
AST (SGOT) (U/L)	28	18	8–137
ALT (SGPT) (U/L)	35	23	16–134
ALK Phosphatase (U/L)	21	14	8–92
GGTP (U/L)	2	3	0–12
Total Bilirubin (mg/dl)	0.1	0.1	0–1.3
Urea Nitrogen (mg/dl)	41	48	13–54
Creatinine (mg/dL)	0.3	0.2	0–0.8
BUN/Creatinine ratio	137	240	
Phosphorous (mg/dl)	3.8	3.7	3.2–7.2
Glucose (mg/dL)	82	100	55–119
Calcium (mg/dL)	9.3	12.8	5.2–11.3
Magnesium (mEq/L)	2.7	1.1	
Sodium (mEq/L)	147	145	120–165
Potassium (mEq/L)	4.1	3.6	
NA/K Ratio	36	40	
Chloride (mEq/L)	118	114	92–128
Cholesterol (mg/dl)	125	19	86–189
Triglycerides (mg/dl)	34	90	10–96
Amylase (U/L)	166	213	244–858
Lipase (U/L)	101	28	
CPK (U/L)	496	423	333–1964
**Complete Blood Count**			
WBC (10^3^/μL)	10.8	4.6	3–43
Hematocrit (%)	29	29	22–64
**Differential (10^3^/ μL)**	**Absolute [%]**		
Neutrophils	5.62 [56]	2.9 [63]	0.6 – 37.4
Bands	0	0	
Lymphocytes	3.67 [34]	0.92 [20]	0.9–13.1
Monocytes	0	0.14 [3]	0–1.6
Eosinophils	1.51 [14]	0.55 [12]	0–5.1
Basophils	0	0.1 [2]	0–1.5

**FIGURE 2:**
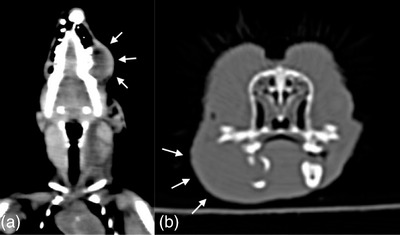
Initial coronal (a) and transverse (b) CT images of the hedgehog. There is a soft tissue swelling that is hypodense relative to the lateral aspect of the left maxilla. The soft tissue swelling is invading the left hemimandible and causing disruption of the underlying bony structure

The hedgehog was premedicated with butorphanol (0.3 mg/kg intramuscular [I.M.], Torbugesic; Zoetis Manufacturing and Research Spain, Girona, Spain) and midazolam (0.4 mg/kg I.M.; West‐Ward Pharmaceuticals, Eatontown, NJ USA). Anaesthesia was mask induced and maintained with isoflurane gas (0.5%) and oxygen (1 L/min). CT scan showed a large soft tissue mass invading the left hemimandible causing underlying cortical disruption (Figure 2). The hedgehog recovered and was discharged with meloxicam (0.5 mg/kg, every 12 h, orally [P.O.], Metacam; Boehringer Ingelheim Animal Health USA Inc., Duluth, GA USA), trimethoprim‐sulfa (33 mg/kg, every 12 h for 14 days, P.O., Sulfatrim Pediatric Suspension; Pharmaceutical Associated Inc., Greenville, SC USA) and Oxbow Carnivore Care (Oxbow Animal Health, Omaha, NE USA).

The hedgehog returned 6 days later for tumour excision and partial left hemimandibulectomy. The hedgehog was premedicated and anaesthetized following previous anaesthetic protocols. An intravenous catheter was placed in the right cephalic vein. The hedgehog was given warm lactated Ringer's solution (1.5 ml/h, intravenously [IV]) intraoperatively. The gingival mucosa was elevated caudal to the left canine and the soft tissues were elevated surrounding the mass. The left mandible was resected caudal to the left canine and cranial to the left ramus. Subcutaneous tissues and the skin were closed with 5‐0 Vicryl in a continuous suture pattern and interrupted suture pattern, respectively. All tissues were submitted for histopathology. Post‐operatively, the hedgehog was administered hydromorphone (0.14 mg/kg, every 6 h, IV and subcutaneously [S.Q.]; Akorn Inc., Lake Forest, IL USA), meloxicam (0.5 mg/kg, every 12 h, P.O.), and warm lactated Ringer's solution (1.5 ml/h, IV). The hedgehog was discharged the following day with meloxicam (0.5 mg/kg, every 12 h, P.O.), tramadol (10 mg/kg, every 6–12 h as needed [P.R.N.], P.O.; Epicur Pharma, Mount Laurel, NJ USA), and Oxbow Carnivore Care with Harrison's Recovery Formula (Harrison's Bird Foods, Brentwood, TN USA).

Histopathology of the excised tissue revealed an infiltrative, unencapsulated neoplasm with extensive areas of necrosis, haemorrhage and inflammation. Tumour cells were elongated with indistinct cytoplasmic borders, small amounts of fibrillar eosinophilic cytoplasm and an elongated cell nucleus with a stippled chromatin pattern and indistinct nucleoli. The mitotic index was 4–8 per high‐power field. These findings were consistent with an incompletely excised spindle cell sarcoma. The owners were warned of a high reoccurrence risk. Despite the paucity of effective chemotherapy protocols in hedgehogs with spindle cell sarcoma, carboplatin therapy (8 mg, every 3 weeks, IV; Intas Pharmaceuticals LTD, Pharmez, Ahmedabad, India) was recommended.

Over the course of 9 weeks, the hedgehog received three doses of carboplatin, which were well‐tolerated, and the animal gained 36 g. However, 2 months after beginning carboplatin therapy a firm swelling was noted overlying the ventral aspect of the remaining left mandible, consistent with tumour regrowth. At this time, the chemotherapeutic agent was switched to lomustine (2 mg, every 2 or 3 weeks, P.O.; Stokes Pharmacy, Mount Laurel, NJ USA). Over the course of 8 weeks, the hedgehog received 5 doses of lomustine which decreased tumour size.

**FIGURE 3:**
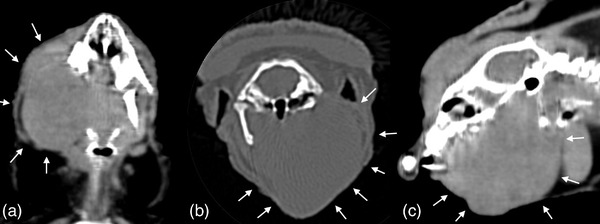
Follow up dorsal (a), transverse (b) and sagittal (c) CT images of the hedgehog. There is a progressively larger soft tissue mass overlying the left mandible. The mass is occluding the airway, displacing the left globe rostrally and dorsolaterally, and causing lysis of the underlying mandibular bone

Approximately 6 months after beginning chemotherapy the tumour increased in size and was noted to invade the oral cavity, partially obstructing the pharynx. The hedgehog also developed a deep corneal ulcer of the left eye. To assess tumour invasiveness a CT scan was performed which showed a progressively larger mass with destruction of the underlying mandibular bone. The mass extended caudoventrally beyond the caudal aspect of the larynx, occluding the airway and causing left exophthalmos, nasolacrimal duct obstruction and exposure keratitis (Figure 3). Given these findings, chemotherapy was discontinued, and palliative external beam radiation was recommended.

The hedgehog was administered a total dose of 8 Gy (400 cGy per treatment) in four fractions over 4 weeks. To facilitate therapy, the hedgehog was anaesthetized following previous anaesthetic protocols and intubated with a 14 g catheter. Devitalized corneal epithelium of the left eye was debrided under anaesthesia and hairs ventral to the left eyelid were pulled as needed. At home, the hedgehog was given oflaxacin (1 drop, every 6 h, left eye [O.S.]; Bausch & Lomb Inc., Tampa, FL USA), genteal eye lubricating gel (1 drop, P.R.N., O.S.; Alcon Laboratories Inc., Fort Worth, Texas USA) and meloxicam (0.1 mg/kg, every 24 h, P.O.). Over the course of a month the keratitis improved, the corneal ulcer healed and the tumour decreased in size.

**FIGURE 4:**
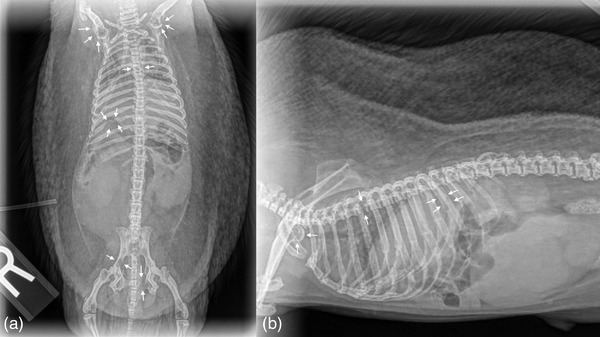
Follow‐up ventrodorsal (a) and right lateral (b) full body radiographs of the hedgehog. There are variably sized lytic lesions within the proximal humeral metaphyses and humeral heads, the body of T5, right ileum, left pubis and ribs (most prominent at the right 11th rib)

**FIGURE 5:**
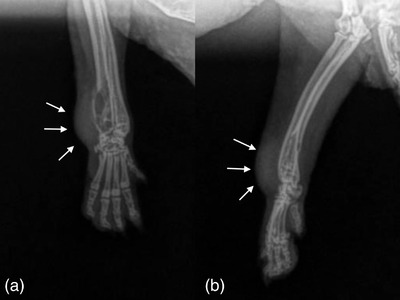
Dorsopalmar (a) and mediolateral (b) radiographs of the right antebrachium and manus of the hedgehog. There is a lytic, expansile lesion with an adjacent dorsolateral soft tissue mass effect overlying the right distal antebrachium

Despite initial improvement, about a month later the hedgehog became acutely non‐ambulatory paraparetic. Upon re‐evaluation, the tumour was larger and displacing the tongue laterally, there was a firm nodule on the lateral aspect of the right carpus, and the urinary bladder was large and firm. Under general anaesthesia blood and urine was collected, fine needle aspirate of the right carpal nodule was obtained and full body and right forelimb radiographs were performed. Radiographs showed variably sized lytic lesions within the proximal humeral metaphyses and humeral heads, body of T5, right ilium, left pubis and ribs (Figure 4). A polyostotic osseous lesion with adjacent dorsolateral soft tissue swelling was noted within the right distal antebrachium (Figure 5). Bloodwork revealed mild anaemia, hypercalcemia, and azotaemia most likely secondary to a paraneoplastic syndrome and post‐renal azotaemia (Table 1). Urinalysis was normal. Cytology of the carpal nodule was consistent with a spindle cell neoplasm. Due to evidence of metastatic neoplasia and poor prognosis the hedgehog was humanely euthanized.

## 2 DISCUSSION

Neoplasia is very common in hedgehogs, with retrospective studies reporting prevalence between 29% and 53% in study populations (Done et al., 1992; Raymond & Garner, 2001; Raymond & White, 1999). Within one study population, 85% of tumours were classified as malignant (Raymond & Gardner, 2001). It is not uncommon to find multiple tumour types with multi‐organ metastasis in hedgehogs (Done et al., 2007; Higbie et al., 2016; Matute et al., 2014; Mikaelian & Reavill, 2004; Papadimitriou et al., 2014; Ramos‐Vara, 2011; Raymond & White, 1999; Tsai et al., 2016; Wellehan et al., 2003). Of the tumour types, epithelial tumours are most common, followed by round cell and spindle cell tumours (Raymond & Gardner, 2001). Oral spindle cell tumours can be highly invasive causing facial swelling, nasal and mandibular deformation, inspiratory dyspnoea, and exophthalmia (Del Aguila et al., 2019).

Due to their locally invasive nature, spindle cell tumours, among other tumour types, are often surgically excised in hedgehogs (Couture et al., 2015; Del Aguila et al., 2019; Done et al., 2007, 1991; Finkelstein et al., 2008; Fukuzawa et al., 2004; Higbie et al., 2016; Koizumi & Kondo, 2019; Martin & Johnston, 2006; Phair et al., 2011; Wellehan et al., 2003; Wozniak‐Biel et al., 2015; Spugnini et al. 2008). Despite successful surgical excision, tumour regrowth can occur. In this report, the spindle cell tumour was incompletely excised with a partial hemimandibulectomy. After regrowth, it was presumed to have metastasized to the thoracic vertebrae, causing spinal cord compression and consequent urinary bladder dysfunction and hindlimb paraparesis. There are other reports of sarcomas compressing or invading the central nervous system and causing neurological deficits in hedgehogs (Delgado et al., 2017; Ogihara et al., 2017; Peauroi et al., 1994).

Chemoradiotherapy (CR) is commonly used for tumour management in canines and felines (Rejec et al., 2015). To date, there are no established CR protocols for hedgehogs and methods are often extrapolated from canine and feline protocols. There are two separate reports of surgical excision and follow‐up radiation therapy (RT) for management of a C‐cell thyroid carcinoma in an African hedgehog and an amelanotic melanoma in a Lesser Madagascar Hedgehog Tenrec (*Echinops telfairi*) (LaRue et al., 2016; Harrison et al., 2010). There is also a single report of electrochemotherapy for management of an oral squamous cell carcinoma in a hedgehog with intralesional bleomycin and trains of biphasic electric pulses (Spugnini et al., 2018). In this report, the hedgehog was given intravenous carboplatin and oral lomustine, followed by RT once the tumour re‐grew. CR was well tolerated and effective in decreasing tumour size in this hedgehog initially, but overall efficacy is uncertain as evidence of metastatic disease led to humane euthanasia.

CR in this report was challenging, primarily due to small patient size and lack of established protocols in hedgehogs. Vascular access was lost during a carboplatin treatment, forcing some of the dose extravascular. Additionally, vascular access could not be obtained during one visit, so the hedgehog needed to return the following week which prolonged therapy.

Detecting evidence of metastatic disease can also be difficult in hedgehogs as metastatic pulmonary nodules can be missed radiographically due to artifact of summating quills. It is important for the clinician to understand that a physical examination under general anaesthesia with complete blood count, serum biochemistry, urinalysis, thoracic radiographs, abdominal ultrasound and fine needle aspirate and cytology are necessary for complete neoplasia workup (Heatley et al., 2005).

This report illustrates that partial hemimandibulectomy and CR for a spindle cell sarcoma could improve long‐term prognosis in a hedgehog. Both treatment modalities were well‐tolerated, but more studies are needed to establish efficacy and specific protocols for the hedgehog patient. This study also highlights the reality that more complete local tumour control, by achieving clear surgical margins initially, will afford better long‐term outcome. If complete tumour excision is not possible and positive margins are found, adjuvant therapy consisting of immediate RT may result in better local tumour control and decrease risk of metastasis. Furthermore, following RT, re‐biopsy of the area could have been performed to assess response to RT, much like is done with human patients (American Society for Therapeutic Radiology and Oncology Consensus Panel, 1999). If considering surgical and medical management, owners should be warned of tumour reoccurrence and the effect this has on survival and prognosis.

## ETHICS STATEMENT

The authors confirm that the ethical policies of the journal, as noted on the journal's author guideline page, have been adhered to. No ethical approval was required as this is a case report with no original research data.

## AUTHOR CONTRIBUTION

Andrew Lacqua – Conceptualization, Investigation, Project administration, Resources, Supervision, Validation, Visualization, Writing‐original draft, Writing‐review & editing

Rebecca Dreizen – Conceptualization, Investigation, Project administration, Resources, Validation, Visualization, Writing‐original draft, Writing‐review & editing

Peter Helmer – Conceptualization, Supervision, Visualization, Writing‐review & editing

### PEER REVIEW

The peer review history for this article is available at https://publons.com/publon/10.1002/vms3.738.

## Data Availability

Data sharing not applicable to this article as no datasets were generated or analysed during the current study
